# The Use of Structured Professional Judgement: A New Way to Understand and Assess Bite Risk from Dogs

**DOI:** 10.3390/ani16060893

**Published:** 2026-03-12

**Authors:** Todd E. Hogue, Helen Howell, Ann Baslington-Davies, Daniel S. Mills

**Affiliations:** 1 School of Psychology, Sport Science and Wellbeing, University of Lincoln, Brayford Pool, Lincoln LN6 7TS, UK; hhowell@lincoln.ac.uk; 2 Department of Life Sciences, School of Natural Sciences, University of Lincoln, Brayford Pool, Lincoln LN6 7DL, UK; 15613067@students.lincoln.ac.uk (A.B.-D.); dmills@lincoln.ac.uk (D.S.M.)

**Keywords:** dog, dog bites, aggression, human directed dog aggression (HDDA), risk, risk assessment, structured professional judgement (SPJ), professional practice

## Abstract

Dog bite incidents constitute significant public health, animal welfare, and economic concerns with substantial physical and psychological consequences for victims. Despite legislative responses, research indicates that breed-focused interventions are ineffective in reducing dog bite risk. Human behaviour, caregiving practices, and environmental context all play central roles in the expression of human-directed canine aggression. Current methods of assessing dog bite risk remain largely unstructured, dog-centred, and reliant on subjective judgement and provocative behavioural testing. These approaches exhibit limited predictive validity and poor reliability, and are vulnerable to bias, raising serious concerns for public safety, judicial fairness, and animal welfare. Comparable challenges in human violence risk assessment led to the development of an evidence-based structured professional judgement (SPJ) assessment framework, which combines empirical risk factors with individualised case formulation and dynamic risk management. An SPJ framework for dog bite risk would ensure the systematic consideration of empirically supported static and dynamic risk factors relating to the dog, caregivers, and related environmental conditions, while supporting the development of targeted risk reduction strategies. Conclusion: Developing an SPJ approach offers a more scientifically grounded, ethically defensible, and prevention-focused method for managing dog bite risk, with potential benefits for public safety, animal welfare, and professional practice.

## Simple Summary

Dog bite incidents constitute a significant public health, animal welfare, and economic concern, with rising incidence in the United Kingdom and substantial physical and psychological consequences for victims. Despite legislative responses, the evidence indicates that breed-focused interventions are ineffective in reducing national dog bite risk. The current methods of assessing human-directed dog aggression (HDDA) are not sufficiently comprehensive and fail to include sufficient breadth of environmental, contextual and owner factors. Assessments need to be more evidence-based in their understanding and management of risk. Clinical and forensic psychology uses structured professional judgement (SPJ) as an effective conceptual framework to assess and predict complex risk decisions related to violence and suicide. Specialist structured professional judgement (SPJ) models, specific to dog bite risk, should be developed to more accurately and effectively assess and manage risks associated with dog bites to humans.

## 1. Review of the Problem

### 1.1. Dog Bites and Their Prevention: A Societal Issue

Dog bite incidents cause both physical and psychological harm to victims at significant economic cost [1], damage to the dog–human relationship, and negative perceptions of dogs by society [2]. It is therefore not surprising that this topic is also of political interest [3]. Unfortunately, in the UK, information on the frequency of dog bite incidents with health impacts is only available through hospital emergency admission data, which includes bites and strikes, while information on community incidents is only available through police data collected by different police forces and is difficult to access. According to hospital data, dog bites and strikes in the UK continue to rise year on year [4]; police forces in England and Wales recorded 30,539 offences where dogs had injured a person or assistance dog in 2023 [5]. A sharp increase in dog-related fatalities in 2023 prompted the UK government to add a fifth breed to the list of prohibited dogs defined in Section (1) of the Dangerous Dogs Act (1991) [6] (DDA), purportedly in the interests of public safety. Under this piece of legislation, it is a criminal offence to own one of the five types specified in the Act without a certificate of exemption. The specified types are the Japanese Tosa, Fila Brasiliero, Dogo Argentino, Pitbull terrier type, and, from 1 February 2024, the American Bully XL type. For a dog to be granted a certificate of exemption it must be neutered, microchipped, have third-party insurance, and be walked on a lead and muzzled at all times when in public.

The DDA has been heavily criticised by a variety of stakeholders for not being an effective way to reduce dog bites or fatalities. The Royal Kennel Club, Royal Society for the Prevention of Cruelty to Animals (RSPCA), and the Scottish SPCA, along with several other organisations including the British Veterinary Association, were collectively represented by the Dog Control Coalition, arguing that breed-specific legislation is ineffective at protecting public safety [7]. Following a continued rise in dog bite fatalities and dog bite incidents in the UK in 2024, the RSPCA, amongst others, called for a broader approach to dog bite prevention focusing on responsible pet ownership, and the WOAH argues similar points internationally [8]. Studies conducted globally support the view of the coalition that breed-specific legislation does not reduce dog bite rates in society [9,10,11,12,13]. In the UK, a study commissioned by the Department for Environment, Food and Rural Affairs (DEFRA) to investigate measures to reduce dog attacks concluded that human behaviour and environmental influences are key factors in the expression of human-directed aggression in dogs [14]. Alternative legislative measures for the prevention of dog bites have been taken elsewhere, including in the city of Calgary, where a responsible pet ownership bylaw focusing on dog licencing, the responsible management of dogs, and educative interventions was implemented in 2021. Dog bite incidents in the city reduced following the implementation of this policy [15].

Educational dog bite prevention strategies have been reported as having a moderate effect on increasing children’s safe behaviours around dogs in the short term [16,17]. However, they do not appear to change the long-term behaviour of children in the presence of dogs or the behaviour of parents in the presence of a dog not known to them [18]. It is suggested [19] that education alone is insufficient, since the multifactorial nature of aggressive behaviour in dogs requires a holistic approach that acknowledges how the behaviour of dogs is the output of an interactive system involving environmental, biological and management factors that recognises the interplay between the dog, the owner, and wider physical and social environments. Thus, focusing on specific features like the breed of dog or specific triggers is a gross oversimplification for the effective management of the risks associated with dog bites, whether that be related to reducing the likelihood or severity of an injury or both. For example, victims of bites from large, powerful dogs are more likely to attend and be admitted to hospital because of the severity of the bite due to size. NHS hospital data only records hospital admissions, therefore providing a biased perspective on the likelihood of a bite being from a large dog breed due to the way the data is collected. This is unhelpful given that we wish to develop strategies to reduce all dog bites, not just those leading to hospital admission.

The cost of dog bite hospital attendance and admissions for NHS England during the period 2017–2018 has been estimated at some £70 million [20]. Given the size of the financial burden and the reported physical and psychological damage to victims of dog bite incidents [21], it seems critical that more focus be given to developing a comprehensive, evidence-based assessment of dog bite risk. Such evidence-based assessments would not only identify high-risk dogs, owners and environments, but are also critical for the development of effective strategies to prevent dogs using aggressive behaviours towards humans.

### 1.2. Dog Bites: A Scientific Perspective on the Quality of Evidence of Risk Factors

For successful prevention strategies to be developed, it is essential to identify factors that may increase the risk of human-directed dog aggression (HDDA).

Studies of dog bite incidents and aggression tend to be retrospective in nature, with many study populations drawn from the medical records of bite victims [22,23,24], clinical behaviour or veterinary records [25,26], or surveys of dog owners [27,28]. Despite there being a significant amount of research on dog bite injuries, which provides useful insights into what is clearly a serious public health issue, systematic research of potential risk factors from unbiased samples is very limited. Newman and colleagues [28,29] applied an approach taken from evidence-based medicine for classifying the strength of evidence from the research literature and were only able to identify unrobust or, at best, limited evidence for a few risk factors for HDDA. They identified moderate and sometimes conflicting evidence for neutering, heritability, some husbandry practices, and children in the home as risk factors, with most studies having methodology and design weaknesses. Another complicating factor affecting research in this area this is that the definition of aggression and the classification of dog bites in scientific research vary between studies and lack consistency [30,31]. This is not an issue that is unique to the field of animal behaviour; in human psychology, the definition and quantification of aggressive behaviour have proven problematic in mental health research [32]. Poor definitions present challenges when evaluating research around the subject of aggression and can be further compounded by a lack of consistency within the scientific literature concerning the target of such aggression (e.g., people in general versus owners, strangers, or children), the definition of this target group (e.g., the definition of a child), and the reference populations used in calculations of relative risk. HDDA is discussed both in general terms and in relation to specific sub-populations (dogs who have not bitten or dogs who have not bitten a specific target group) [33]. Without the clear definition of all factors of relevance, research intended to prevent the occurrence or severity of dog bites is of much more limited value.

Most work on dog bite risk reduction focuses on owned dogs, and legislation in many countries holds the owner responsible for the actions of their animals [6]. They are also the primary vehicle through which any recommendations for managing risk will often be implemented. This might, for example, include actions like undertaking dog management and behaviour training for the owner or the use of a muzzle for the dog. This also includes the implementation of specific management strategies aimed at both personal and public safety. Thus, the identification of elements of owner characteristics and interactions with their dogs that may influence HDDA is particularly important from a risk management perspective [28,30] as it highlights, in particular, the opportunities for risk reduction through owner behaviour change [34].

Two recent systematic reviews have attempted to update the knowledge base of human-directed aggression from dogs through the systematic evaluation and synthesis of research relating to the influence of the early experience of dogs [35] and human-related factors [33] on HDDA. Unlike previous reviews, these studies considered not only the quality of the study design, but also evaluated the robustness of the evidence by considering the consistency of evidence across less powerful designs. Nonetheless, they continue to highlight the lack of studies with robust methodologies, particularly where causality can be inferred. While these reviews update the evidence base regarding what is known about HDDA, they do not speak directly to the issue of the assessment of dog bite risk.

### 1.3. Dog Bite Risk Assessment: Ethical, Legal and Practical Consequences of Poor Assessment

The assessment of dog bite risk is carried out across a range of contexts, including criminal proceedings following a dog bite incident, the pre-adoption assessment of shelter dogs, and in the context of social care, where children and dogs may share a home. The development of effective and accurate dog bite risk assessments has clear legal and ethical implications. In addition, judgements made with respect to “dangerous dog” matters have serious implications both legally (related to public safety) and ethically (related to the lifestyle choice and quality for both owners and dogs, in addition to potentially demanding the euthanasia of the dog involved). Risk assessments tendered as evidence in legal forums should be relevant to the legal issue in dispute, focused on the specific dog/situation concerned, evidence-based, and conducted by trained and experienced evaluators. Unfortunately, there is a risk of a much more ad hoc approach as the professionals involved in assessment are working in an unregulated industry [36]. This lack of consistent, structured and evidence-based assessments is of concern, both as a public safety and an animal welfare issue, quite apart from raising issues of legal responsibility and fairness for the judiciary [37].

In the context of assessing risk for legal judgements in the UK, risk assessments are typically undertaken by individuals who self-identify as “experts” in the area of dog behaviour. They may rely on the use of practical assessment batteries that may take the form of provocative tests. These are frequently carried out in a controlled environment, which is vastly different to any dog-bite incident or the normal living conditions of the dog. Observations and interpretations of the dog’s behaviour under these conditions are then translated to the real world. Examples of provocative tests include presenting the dog with stimuli, such as a real or artificial dog or childlike doll; removing a food bowl from the dog when it is eating, sometimes with an artificial hand; or exposing the dog to a startling noise. The dog may be presented with the stimuli one after the other over a relatively short period of time. This process implies that the evaluator believes that the dog’s behavioural responses during the tests are in some way related to the overall behavioural tendencies of the dog [37]. There is not even a requirement for standardisation of the provocative activity or a requirement that the approach used by different assessors or in different assessments is standardised. Thus, the assessment relies heavily on the subjectivity of the individual assessor and their use of an unstructured judgement of what is critical and predictive of risk in the situation. The inherent subjectivity of risk assessments made using an unstructured judgement approach is a major issue likely to result in poor reliability and predictive validity [38]. These approaches do not meet the basic requirements for the valid assessment of behavioural predispositions in dogs [39], and their predictive value in other contexts has been heavily criticised [40].

In addition, in the UK, for example, individual assessors do not need to be trained in the assessment of risk, nor is there a requirement for specific factors to be assessed or considered during this process. Indeed, in the UK, there are currently no registration requirements or standards expected by the Court as a minimum for expert evidence with respect to dog bite risk to be admissible. The decision-making process is also vulnerable to the availability heuristic; for example, recent high-profile fatal incidents involving breeds of dog that are frequently negatively portrayed in the media may result in an individual overestimating any risk posed by an individual dog of a similar breed type being assessed [41].

Within the shelter environment, the use of behavioural assessments has been common for decades, though types of assessment, the behaviours assessed, and consistency in application vary greatly [42]. These behaviour tests are often used with the hope that they will help to establish whether a dog is suitable for adoption or whether it would pose a danger to the public; to match the dog with a suitable adopter; and/or to identify problematic behaviours that would benefit from behavioural intervention pre and/or post adoption to reduce the risk of relinquishment or harm. However, behavioural assessments carried out in a shelter environment prior to adoption have very limited predictive value in terms of a dog’s behaviour in a home environment post adoption [43]. Even where assessment tools have been demonstrated to have predictive validity, results have been shown to differ across different test environments [44]. Many situations that are likely to arise in a home environment do not lend themselves to comprehensive testing in a shelter environment. Further, animal shelters often use non-validated assessment protocols developed in-house. Consequently, these assessment tools often lack standardisation and have not been scientifically evaluated for their quality [45]. This raises the question of the likely value of this approach to assessment.

It has been suggested that, rather than exposing shelter dogs to provocative testing and relying on evaluations that are not evidentially supported, it would be more beneficial to focus on thorough history-taking at the point of intake and observing a dog’s behaviour over a period of time when engaged in activities the dog would be exposed to in a home environment like walking, playing and socialising [40]. This approach is currently taken by some rescue organisations; however, there is an absence of standardised, evidence-based guidance applied across organisations. Some rehoming organisations utilise a semi- or quasi-standardised matching program where potential adopters are matched to specific dogs based on their individual needs, wants and lifestyle [34]. However, one study revealed that out of thirty-seven factors identified through screening that were thought to be important by rehoming staff, only four were evidentially supported as being associated with risk to human safety [46]. The importance of this is easy to overlook until it is appreciated that the results of such tests may be routinely used in shelters to make life or death decisions for the dogs concerned, due to the limited resources of the shelter.

Quite apart from the ethical issue of the use of inappropriate information for euthanasia decision making, there is also a potentially high emotional burden on animal shelter staff associated with behavioural euthanasia decisions. The euthanasia of healthy animals has been found to be more stressful for staff than the euthanasia of sick or injured animals [47,48]. Providing structured, evidence-based guidelines for staff responsible for the assessment of the risk that a dog may pose to human safety may not only help to improve decision making, but also to reduce the burden faced by individual assessors, offering some buffer against euthanasia-related stress [43].

## 2. Learning from Other Fields

### Forensic Violence Risk Assessment: Lessons from the Human Field

Assessing the risk of human violence is a key element in legal decision-making, particularly in matters such as sentencing and bail. In the UK, the use of risk assessment tools is considered best practice within forensic mental health and criminal justice, where these instruments are commonly applied [49,50]. Traditional assessments relied on clinicians’ intuition and professional experience to judge “dangerousness”; however, this approach has been widely criticised for its subjectivity, unreliability, and lack of accountability [51]. Today, there is broad agreement that unstructured clinical judgement alone is insufficient and lacks validity for assessing violence risk [52].

To address the shortcomings of purely subjective assessments, actuarial risk assessment tools were introduced. Notable examples include the Statistical Information on Recidivism Scale [53] and the Level of Service Inventory [54]. These tools employ structured, formulaic methods to quantify risk factors associated with negative outcomes, such as reoffending, and convert them into estimated recidivism rates [55]. Actuarial approaches provide objective, data-driven assessments and have demonstrated strong predictive validity [56,57,58]. Nevertheless, they primarily focus on static risk factors—attributes that do not change over time—prompting criticism that they fail to account for individual complexities and the prospect of positive change through intervention [59,60].

In response, structured professional judgement (SPJ) methods have been developed. These approaches blend research-based risk factor identification with specific case information and professional expertise, allowing for the inclusion of both static and dynamic factors. Dynamic factors, such as substance misuse, can vary with time and intervention [61]. This flexibility is especially important for risk management strategies, as SPJ enables the ongoing evaluation of individuals’ risk profiles in light of changing circumstances [61,62,63]. Practitioners using SPJ can distinguish between a person’s overall risk status (relative to others) and their current risk state (based on recent changes in their life) [64]. Originally intended to support decision-making on risk levels, structured violence risk assessment tools now also inform effective risk management and reduction strategies.

Comparative research between actuarial and SPJ approaches has produced mixed findings. Some studies suggest that incorporating dynamic factors enhances predictive accuracy for recidivism [65], while meta-analyses indicate that both methods generally exhibit similar levels of validity [66,67].

In the UK, the Risk Management Authority (RMA), an independent non-departmental public body, was established in 2005 under the Criminal Justice (Scotland) Act (2003) with the function of ensuring effective assessment and minimisation of risk. The RMA publishes standards and guidelines for the risk assessment and management of violent and sexual offending. The Risk Assessment Tools Evaluation Directory [68] catalogues risk assessment instruments, both validated and awaiting validation, to assist practitioners in selecting and applying risk assessment tools. The RMA advocates an approach to risk assessment that is evidence based, defensible and proportionate [68].

The SPJ approach is particularly helpful in guiding professionals in the production of risk management plans and identifying intervention opportunities. As such, they are effective in both predicting and potentially preventing the behaviour or incident of concern. It is not surprising, therefore, that the use of SPJ instruments has expanded to other areas where the assessment of risk and risk management are professionally practised. This includes suicide risk [69,70], the allocation of patients to appropriate levels of therapeutic security [71], and the risk of terrorism or extremist violence [72,73]. Indeed, structured professional judgement is considered the best practice approach for assessing terrorism risk [74].

## 3. Discussion

### 3.1. Applying Structured Professional Judgement (SPJ) to the Assessment of Dog Bite Risk

Given the multifactorial nature of human-directed dog aggression, a comprehensive, structured approach considering evidentially supported factors related to the dog, the dog’s caregivers, and the environment in which the dog will be living is likely to be more effective in assessing risk than the heavily dog-focused approach that has been traditionally taken. The use of an SPJ may also help mitigate against some of the concerns associated with the quality of those undertaking assessments and the tools they use.

The assessment of risk in the field of forensic psychology is well established and has been the subject of extensive research. It has been suggested that an interdisciplinary approach, based upon methods used for human violence risk prediction, be developed for the assessment of the risk a dog may pose to human safety [75]. We propose that the structured professional judgement approach is the most appropriate system to apply to dog bite risk assessment. Using an SPJ approach would provide professionals with an improved risk assessment structure. SPJ guidelines would increase the accuracy and consistency of assessments by establishing an assessment structure focused on empirically evidenced risk factors and reducing implicit bias by making assessors attend to all evidence-based risk factors, not just the ones they implicitly think of. It would also potentially allow the systematic assessment of dog bite risk to be tailored to account for the dynamic nature of relevant human and environmental factors. The consideration of both static and dynamic risk factors assists in the identification of risk management strategies. In forensic psychology, there is a responsibility and ethical obligation for clinicians to reduce and effectively manage the risk of future violence [76]. It is suggested that professionals carrying out the assessment of the risk of human-directed dog bites have a similar moral obligation to protect public safety. The use of an SPJ model would provide appropriately trained professionals with evidence-based guidance to not only identify risk factors, but also devise risk reduction strategies with the ultimate goal of dog bite prevention.

To develop an effective and accurate system of predicting and managing dog aggression, there needs to be a fundamental shift in the approach to the problem. The focus must be on developing a genuine, evidence-based system to accurately predict aggressive behaviour within the dog’s normal environmental context. To do this, the field needs to focus on a number of key aspects that any future method should address.

### 3.2. Steps to Developing SPJ Guidelines for Dog Bite Risk

In the current context, it is important to explain how an SPJ system could be developed and then implemented to assess dog bite risk. This process is already well defined in the area of human behaviour prediction [77]. We outline below, in broad terms, the steps required to create and implement such a system to address the current challenges in understanding dog bite risk.

#### 3.2.1. Clearly Define the Behaviour of Concern

The starting point of any SPJ system is to clearly define the behaviour of concern. It is our view that dog bite behaviour should be the focus. If it is dog bite behaviour, then that definition would need to be clear about what constitutes a bite. For example, lunging, growling and dog-on-dog aggression may constitute important dog behaviour, but we would argue do not constitute the behaviour of interest for the SPJ system. We argue that a dog biting a human is what needs to be the first and primary focus for the following reasons:

- Any bite behaviour is of concern because there are the resulting physical injury, psychological harm, and trauma to consider for any bite victims or those observing.
- The action of biting needs to be focused on, as dogs who have bitten in the past, even with minor harm, may be more likely to bite in the future, possibly with a more serious outcome.
- We focus on bite likelihood rather than the severity of the injury, as severity is at least partially related simply to the size and capacity of the dog and less related to the likelihood of future bite occurrence.
- This does not include dog strike or accident-related behaviour or dog-on-dog aggression, and we leave it to others to investigate these behaviours.

#### 3.2.2. Identify the Empirically Based Risk Factors Related to Dog Bite Behaviour

The agreed definition of dog bite behaviour should be used to identify a clear and comprehensive understanding of the empirical evidence supporting the different risk factors. This step needs to consider the existing information and knowledge base related to dog bite risk, including published research information and public or professional opinion. The identified evidence should be evaluated and weighted in terms of the extent that there is empirical support for each identified risk factor. Key considerations for evaluation include the definition of dog bite behaviour evaluated, the robustness of research designs, the application of suitable statistical analyses, and the integration of public and professional perspectives only through rigorous qualitative methodologies rather than relying on isolated personal or social media opinions. In practical terms, this step is likely to be achieved through systematic reviews of the literature and comprehensive qualitative studies of relevant populations.

#### 3.2.3. Consolidate the Knowledge Base into Definable Evidenced Risk Factors

It is anticipated that the research evidence on dog bite risk will use a wide range of terms and definitions to describe different aspects of dog bite behaviour. The defined behaviour being studied and the strength of the of the empirical support and generalisability of the findings is likely to be highly variable [28,33,35]. The first step is to consolidate this into a usable list of risk factors.

The first part of this step is to undertake a thematic analysis of the evidence that is identified to define a comprehensive list of the areas where there is evidence of risk related to dog bites. Once all empirically supported risk evidence has be thematically consolidated, an agreed number of identified risk factors, each supported by empirical evidence, will be identified. This list of risk factors will be comprehensive in that it will include information from all the identified empirically supported evidence. This list then becomes the core of the SPJ system for dogs, similar to the way that the 20 items act as risk factors in the HCR-20 guidance [77,78,79].

#### 3.2.4. Develop Risk Factor Definitions and Guidance

The next step is to develop a definition of each risk factor that encapsulates the focus and theme represented by each factor. This description should be a relatively short textual description of how the risk factor would present in practice. It should be noted that an individual examining an individual dog or event could make a judgement regarding the extent to which a factor fully, partially, or did not apply.

As an example, a risk is likely to be identified related to unmanaged health and medical needs. These have been identified in the literature relating to the increased risk of a dog biting [80,81] and, in our view, this this is an example of an issue that needs to be considered when undertaking an assessment of risk. The risk factor definition might be:

Unmanaged Health and Medical Needs: Consideration should be given to the dog’s general health and any medical conditions, including the presence of pain and discomfort. When health issues are fully managed without any residual effects, this would not apply. However, evaluators should consider the possible side effects of any medications the dog may be taking, residual pain, and the influences on behaviour these may have.

For each of the agreed risk factors, guidance would then be developed relating to the likely observable indicators of the risk factor, the evidence supporting the risk factor, and information relating to how and why the factor might be managed or mitigated. The SPJ risk assessment can be conducted in relation to the current situation, owner and environment or as anticipated relating to a future situation, owner and environment, for example in the case of rehoming.

The development of this resource for each of the identified risk factors including the definitions, research information and guidance would constitute the SPJ tool for dog bite risk.

### 3.3. Use of SPJ Guidelines in Practice

Once SPJ guidelines have been developed and are available for dog bite risk, it is important to ensure that they are used accurately and consistently. To do this, it would be necessary to establish training program, practice standards and a user registration system similar to that in the violence predication area [68]. The development of these governance structures would ensure that there is consistency in the way that SPJ-based assessments are conducted and that the courts and other organisations can be confident in the quality of the assessment being undertaken. Importantly, this should also improve the accuracy of public perception regarding dog bite risk by improving the public understanding of all dog bite risk factors, not just those that tend to be raised in the media, such as breed.

An effective SPJ-based dog bite risk tool should:

1. Include the prediction of dog bites that result in low-grade injury as well as those that lead to serious injury.
2. Include a comprehensive, evidence-based range of risk factors specifically related to dog bite risk.
3. Be able to be used by a range of animal and dog behaviour professionals with appropriate training and demonstrate high intra-rater reliability.
4. Provide an objective way of measuring and evaluating the effectiveness of the SPJ system, including inter-rater reliability and standardised prediction and error rates.
5. Demonstrate high inter-rater reliability between different assessors assessing the same dog/situation.
6. Be able to evidence how effectively it can predict dog bites over identifiable periods of time and to quantify this statistically.
7. Demonstrate predictive validity by being able to identify those animals, owners and situations most likely to present an increased bite risk in the future.
8. Be used to develop, implement and evaluate the effectiveness of risk management plans.

## 4. Conclusions

Future research needs to focus on including the full range of dog bites and not just the more injurious ones [77]. On this basis, a comprehensive suite of potential risk factors needs to be considered, encompassing the animal’s characteristics and life history, social and physical environmental factors, and owner characteristics. In light of practical limitations to study design associated with the standards used in evidence-based medicine (e.g., the use of randomised controlled, blinded studies is infeasible and unethical), alternative approaches need to be considered to evaluate current knowledge. This may include the empirical investigation of the collective opinion of experts and those working with dogs alongside epidemiological studies of risk. Some of these variables are static (e.g., breed, bite history) and others dynamic (e.g., environment, owner factors) and both need to be considered to create an assessment that is both reflective of risk and responsive to change.

Any developed system should then be tested in terms of reliability and predictive validity in a similar way to the development and validation of methods used for predicting and managing human violence risk that has been achieved in forensic psychology. The tool should then be compared with current methods of aggression assessment and judged in terms of its relative predictive accuracy. Such a system should ultimately not be dependent upon a specific ‘expert’, but would demonstrate reliability across a range of different, appropriately trained assessors. The ultimate aim of this evidence-based stream of research would be to develop a structured professional judgement system, with good predictive validity, to facilitate the assessment and management of the risk of aggressive behaviour in dogs, something that is currently sadly absent in this area. The primary aim of developing SPJ guidance for dog bites is to reduce the risk to the public of dog bites and to improve the response of professionals working in the area. A secondary benefit is that it should improve public understanding of the range of dog bite risk factors and that simple, single-cause explanations and interventions are unlikely to be effective in reducing dog bites.

## Data Availability

The original contributions presented in this study are included in the article. Further inquiries can be directed to the corresponding author.
